# Therapeutic drug monitoring with non-anti-TNF advanced therapies in inflammatory bowel disease: current and evolving paradigms

**DOI:** 10.3389/fgstr.2026.1860608

**Published:** 2026-08-04

**Authors:** Faris Chater, Thomas D. Butler, Jimmy K. Limdi

**Affiliations:** 1 Department of Gastroenterology, Northern Care Alliance NHS Foundation Trust, Manchester, United Kingdom; 2 Division of Diabetes, Endocrinology and Gastroenterology, Faculty of Biology, Medicine and Health, The University of Manchester, Manchester, United Kingdom; 3 University of Manchester, Manchester, United Kingdom

**Keywords:** Crohn’s disease, drug clearance, etrasimod, filgotinib, guselkumab, inflammatory bowel disease, mirikizumab, ozanimod

## Abstract

Inflammatory bowel diseases, encompassing Crohn’s disease and ulcerative colitis, are chronic inflammatory disorders of the gastrointestinal tract. The recent expansion in advanced therapy options has not dramatically altered the ceiling of treatment efficacy. Therapeutic drug monitoring (TDM) of the serum drug levels and anti-drug antibodies has the potential to optimise treatment efficacy through dose adjustment. TDM has shown benefit with purine analogues and anti-tumour necrosis factor (TNF) therapy. Less is known about the role of TDM in non-anti-TNF advanced therapies. This review summarises the current evidence base for TDM in non-anti-TNF advanced therapies.

## Highlights

- Despite growing data documenting the associations of drug concentrations and ADA in non-anti-TNF antibodies with outcomes, data are not currently sufficiently robust to recommend TDM in non-anti-TNF advanced therapies.
- The documented ADA rates are lower in non-anti-TNF advanced therapies, which reduces the potential utility of ADA testing for these therapies.
- There are insufficient data to recommend proactive or reactive TDM in non-anti-TNF advanced therapies.

## Background

Inflammatory bowel diseases (IBDs) encompassing Crohn’s disease (CD) and ulcerative colitis (UC) are chronic, inflammatory, immune-mediated conditions of the gastrointestinal tract with a myriad of extraintestinal manifestations, which are often associated with a negative impacts on quality of life (1). Patients frequently require long-term treatment with advanced therapies that abrogate immunoinflammatory pathways. Modern paradigms advocate treating beyond symptoms to the target of clinical and endoscopic remission, aiming to reduce long-term use of corticosteroids and to prevent long-term complications and disability (2, 3). Anti-tumour necrosis factor (TNF) therapies remain an important advanced treatment option in IBD, although the therapeutic choice is increasingly individualised according to the disease phenotype, comorbidities, and patient factors. However, it is well recognised that primary non-response affects up to a third of patients, with loss of response in nearly 50% of initial responders by 12 months and a further 20% experiencing loss of response annually thereafter (4, 5). Despite the advent of novel advanced therapies including anti-integrin, anti-p40 [interleukin 12 (IL-12) and IL-23], anti-p19 (IL-23) monoclonal antibodies, and oral small molecules [inhibitors of the Janus kinase (JAK) pathway and sphingosine-1-phosphate (S1P) receptor modulators], improvement in signs and symptoms and achievement of mucosal healing occur in distinctly fewer than 50% of treated patients, highlighting a therapeutic ceiling and the need to optimise the use of existing treatments (1).

The significant and sobering negative impacts of suboptimal biologic use on disease burden and healthcare expenditure underscore the development of therapeutic drug monitoring (TDM). TDM offers a strategy to guide precision therapy by measuring the serum drug levels and anti-drug antibodies (ADAs) with the aim of maximising efficacy, minimising toxicity, and informing when to adjust or switch treatment (6). Current approaches to TDM include reactive TDM, where the drug levels and ADAs are measured following suspected treatment failure (either primary or secondary loss of response), and proactive TDM, where monitoring is performed at predefined time points to optimise drug exposure before clinical deterioration occurs (1, 7).

The most robust evidence base for TDM in IBD exists for thiopurines (8) and anti-TNF therapies (9). In contrast, TDM for non-anti-TNF advanced therapies is less well established, wherein inherent complexity associated with the pharmacological properties of newer biologic and small-molecule therapies limit broader generalisations. This review summarises the current evidence for TDM in non-TNF advanced therapies for IBD.

## Methods

This article is a narrative review. Relevant literature was identified through searches of PubMed/MEDLINE and Embase from database inception to April 2026 using combinations of terms relating to IBD, TDM, pharmacokinetics, trough levels, and individual drug names. Peer-reviewed English-language articles were eligible, with primary data preferentially included. The included studies were evaluated for topic relevance and methodological robustness. Studies were selected for inclusion if they addressed serum drug concentrations, ADA, exposure–response relationships, dose optimisation, and proposed therapeutic thresholds. The key pharmacological characteristics of the non-anti-TNF advanced therapies are summarized in **Table 1**.

**Table 1:** Key characteristics of non-anti-tumour necrosis factor (TNF) advanced therapies in inflammatory bowel disease (IBD).

Drug	Indication	Mechanism of action	Pharmacokinetics (PK)	Pharmacodynamics (PD)	Half-life	Immunogenicity
Vedolizumab	Ulcerative colitis Crohn’s disease	α4β7 integrin antagonist	Clearance increased with severe obesity, low albumin, and high inflammatory burden; low immunogenicity	Gut-selective inhibition of lymphocyte trafficking	~25.5 days	Low (<5%)
Ustekinumab	Ulcerative colitis Crohn’s disease	IL-12/23 inhibitor targeting the p40 subunit	Clearance influenced by body weight, albumin, CRP, and prior anti-TNF exposure; low immunogenicity	Inhibition of IL-12- and IL-23-mediated inflammatory pathways	~14.9–45.6 days	Low (2.3%)
Risankizumab	Ulcerative colitis Crohn’s disease	IL-23 inhibitor targeting the p19 subunit	Clearance increased with high body weight and low albumin and affected by female sex, CRP, and prior advanced therapy exposure, without clinically relevant impact on dosing	Inhibition of IL-23-mediated inflammatory pathways	~21–28 days	Low (2%–4%)
Mirikizumab	Ulcerative colitis Crohn’s disease	IL-23 inhibitor targeting the p19 subunit	Clearance influenced by body weight and albumin, without clinically relevant impact on dose adjustment	Inhibition of IL-23-mediated inflammatory pathways	~9.5 days	ADA 23%; limited clinical impact
Guselkumab	Ulcerative colitis Crohn’s disease	IL-23 inhibitor targeting the p19 subunit	Clearance influenced by body weight, albumin, CRP, age, sex, and prior biologic failure, without clinically meaningful impact on dose adjustment	Inhibition of IL-23-mediated inflammatory pathways	~16.5 days	Low (1.4%–5%)
Tofacitinib	Ulcerative colitis	Pan-JAK inhibitor with preference for JAK1 and JAK3	Oral small molecule with linear PK; rapid absorption with peak concentration at ~1 h; plasma levels unaffected by disease severity and stable over time	Inhibition of JAK-mediated cytokine signalling pathways	~3 h	None (small molecule)
Filgotinib	Ulcerative colitis	Selective JAK1 inhibitor	Oral small molecule; rapidly absorbed with peak concentration at 1–5 h; metabolised to an active JAK1-selective metabolite	Inhibition of JAK1-mediated cytokine signalling pathways	~5 h	None (small molecule)
Upadacitinib	Ulcerative colitis Crohn’s disease	Selective JAK1 inhibitor	Oral small molecule; rapid absorption with peak concentration at 1–2 h	Inhibition of JAK1-mediated cytokine signalling pathways	8–14 h	None (small molecule)
Ozanimod	Ulcerative colitis	S1P receptor modulator targeting S1P1R and S1P5R receptors	Oral small molecule; metabolised to active metabolites; pharmacokinetics influenced by body weight and smoking status, without clinically relevant impact on dosing	Modulation of S1P1 and S1P5 receptor signalling	21 days (parent); 84–117 h (metabolites)	None (small molecule)
Etrasimod	Ulcerative colitis	S1P receptor modulator targeting S1PR1, S1PR4, and S1PR5 receptors	Oral small molecule with linear pharmacokinetics; steady state achieved after ~7 days of dosing	Modulation of S1PR1, S1PR4, and S1PR5 receptor signalling, resulting in dose-dependent lymphopenia	30 h	None (small molecule)

### Therapeutic drug monitoring with vedolizumab

Vedolizumab (VDZ) is a humanised immunoglobulin G1 (IgG1) monoclonal antibody solely targeting the α4β7 integrin expressed on lymphocytes in gut-associated lymphoid tissue, which interacts with mucosal vascular addressin cell adhesion molecule-1 (MAdCAM-1) (10). The α4β7 integrin plays a key role in lymphocyte trafficking to the gastrointestinal tract, and selective blockade of this pathway results in gut-selective inhibition of lymphocyte trafficking (10).

VDZ has a prolonged half-life of approximately 25.5 days, with linear elimination as it binds to the neonatal Fc receptor protecting the antibody from proteolysis (10). Drug clearance is similar for both CD and UC, but is higher in people with severe obesity (>120 kg) and low levels of albumin (<3.2 g/dl) (11). An increased inflammatory burden and the development of neutralising antibodies increase VDZ clearance. Concomitant immunosuppressive drugs (e.g., methotrexate and thiopurines) do not appear to impact VDZ clearance (12–14). Immunogenicity with VDZ is uncommon, which has been reported in 12% of patients who discontinued VDZ (12, 13). In GEMINI trials, the ADA rates of VDZ were low (<4%) (12, 13). Real-world studies also demonstrated low rates of antibody formation (<5%) with both drug-sensitive and drug-tolerant assays, and VDZ ADAs have rarely been the cause of treatment failure (15).

Pharmacokinetic studies have demonstrated that dosing of 8-weekly VDZ saturates more than 95% of the α4β7 receptors on peripheral lymphocytes (16). This might suggest that increasing the concentrations of VDZ when the target receptor is saturated would not impact on augmenting efficacy. A dose-dependent differential binding of VDZ to different T-cell subpopulations has been demonstrated by *in vitro* and *in vivo* studies, suggesting an optimal ‘window’ of exposure (17).

The pivotal GEMINI trials demonstrated the efficacy of VDZ in the induction and maintenance of UC (GEMINI 1) and CD (GEMINI 2) (12, 13) in both anti-TNF-naive and exposed patients. In the induction phase, both trials demonstrated a trend towards incrementally higher rates of clinical response with each quartile increase in VDZ trough concentration. In a *post-hoc* analysis using patient-level data from GEMINI 1, which allows for control of the variables affecting VDZ clearance, such as albumin level and body mass (18), the week 6 and week 52 clinical remission rates were shown to significantly increase with higher week 6 VDZ concentrations, leading to proposed target concentrations >37.1 μg/ml at 6 weeks and >12.7 μg/ml at steady state (after 18 weeks). *Post-hoc* analysis demonstrated that the clinical remission in participants with trough concentration <17 μg/ml was no different from that of placebo. However, the VDZ concentration was a stronger predictor of the week 6 clinical response than the serum albumin level, faecal calprotectin, and prior anti-TNF therapy use (18).

In LOVE-CD, a prospective, non-randomised study on the efficacy of VDZ, the median VDZ concentrations were often, but not always, higher in participants with endoscopic remission across follow-up visits, and a VDZ concentration >10 μg/ml at week 22 was a good discriminator of endoscopic remission at week 26 [area under the curve (AUC) = 0.74].

In ERELATE, a large, retrospective multicentre observational study, a VDZ concentration >30 μg/ml during induction was associated with increased rates of clinical remission at weeks 14 and 52, suggesting that higher induction concentrations (or lower drug clearance) may be important for successful maintenance (19).

In the GEMINI long-term study, participants who withdrew from GEMINI 2 were dose escalated to 4-weekly VDZ, which was associated with clinical remission in 13/57 (23%) at 28 weeks compared with 2/57 (4%) prior to dose escalation (20). Other observational studies also associated higher VDZ concentrations with better outcomes involving a variety of clinical, biochemical, and endoscopic metrics (21, 22), albeit with bias inherent to small observational trials, limiting the generalisability of the results, particularly where VDZ concentration cutoffs are proposed.

The ENTERPRET trial included 278 patients with moderate–severe UC for VDZ dose optimisation (23). It enrolled primary non-responders with a week 5 VDZ concentration <50 μg/ml and randomised to a standard dose or a dose-optimised regimen for the week 6 induction dose (23). It demonstrated that dose optimisation did not improve the clinical or endoscopic response at week 30 (23). However, it could be argued that the likely slower onset of action for VDZ may have diluted the effect of dose optimisation, and the eligibility VDZ concentration cutoff of 50 μg/ml corresponded to the highest quartile 4 VDZ concentration at week 6 in GEMINI 1. Indeed, a recent multicentre study from Canada also found that the induction and post-induction serum VDZ concentrations were not consistently associated with biochemical normalisation in CD and UC (24).

### Dose escalation

Data on the dose optimization of VDZ in patients with low trough levels experiencing loss of response are limited (25). In the GEMINI long-term study, patients on maintenance therapy with loss of response who were dose escalated from 8-weekly to 4-weekly VDZ demonstrated increased clinical remission rates from 4% to 32% (20). However, in a Belgian study of 62 patients with IBD with loss of response, dose optimisation increased the median VDZ trough concentration from 8.8 µg/ml [interquartile range (IQR) = 5.1–13.5 µg/ml] (T0) to 19 µg/ml (IQR = 11.9–22.9 µg/ml) (T1) and 23.1 µg/ml (IQR = 15.5–28.4 µg/ml) (T2) (all *p* < 0.0001), but did not correlate with clinical response (20, 26).

VDZ is now available as a subcutaneous (SC) formulation based on the results of the VISIBLE I [Efficacy and Safety of Vedolizumab Subcutaneously (SC) as Maintenance Therapy in Ulcerative Colitis] and VISIBLE 2 [Efficacy and Safety of Vedolizumab Subcutaneous (SC) as Maintenance Therapy in Crohn’s Disease (CD)] studies. These studies confirm that maintenance therapy with the SC formulation conferred efficacy and safety similar to the intravenous (IV) formulation in patients with UC and CD who responded to IV induction (27, 28). The median serum VDZ level was higher in patients receiving the SC dosing (39.8 µg/ml, 90%CI = 20.8–75.4 µg/ml) *versus* the IV dosing at 32.2 µg/ml (90%CI = 16.5–60.7 µg/ml). The rate of anti-VDZ antibodies was low at 6% in patients receiving either SC or IV VDZ, and the rate of neutralising antibodies was also low at 3% in both groups (27, 28). Notably, the dosing frequency was every other week with SC VDZ compared to q8w with IV dosing, and as such, the SC steady-state trough levels of 34.6 and 30.2 mg/ml in VISIBLE 1 and 2, respectively, cannot be directly compared to the trough levels with IV administration protocols.

Taken together, despite a growing body of evidence suggesting an exposure–efficacy relationship with VDZ, the heterogeneity in the study design, case definitions, and outcome parameters do not enable firm conclusions for clinical practice and routine proactive TDM cannot currently be recommended. Available data suggest that VDZ concentrations of 33–37 µg/ml at week 6, 15–20 µg/ml at week 14, and 10–15 µg/ml during maintenance may be associated with improved outcomes (12, 13, 19, 29).

### Ustekinumab therapeutic drug monitoring in Crohn’s disease

Ustekinumab (UST) is an anti-IL12/IL23 antibody targeting the common p40 subunit and is licenced for the induction and maintenance of remission in moderate to severe CD and UC. It is administered as an IV induction dose at 6 mg/kg followed by 90 mg SC every 8 or 12 weeks. The bioavailability of UST following SC administration is approximately 57%, and the median times to maximum serum concentration after single SC doses of 45 and 90 mg were 13.5 and 7 days, respectively (30).

The half-life of UST varies between 14.9 and 45.6 days (31). Drug clearance is related to differences in body weight, serum albumin level, C-reactive protein (CRP), having failed an anti-TNF treatment, and the presence of antibodies against UST. Patients weighing >100 kg have a median clearance 55% higher than those with weighing ≤100 kg (32). Similarly to VDZ, the addition of an immunomodulator does not appear to significantly influence the concentrations of UST (33, 34).

In the UNITI-1 and UNITI-2 induction trials, higher median serum concentrations were reported 8 weeks after the 6-mg/kg UST dose compared with the 130-mg dose (6.3–6.4 *vs*. 2.0–2.1 μg/ml) (35). UNITI-2 demonstrated higher rates of clinical remission with UST given 8-weekly compared with 12-weekly (33). In the maintenance IM-UNITI trial open to patients who had responded to UST induction in UNITI, steady-state serum levels were reached by week 24 (33). When the 8- and 12-weekly dosing data were combined from IM-UNITI, higher serum UST concentrations were associated with increased rates of clinical remission at week 24, which remained statistically significant in stratified analysis of the 8-weekly, but not for the 12-weekly, dosing group (33). At week 44, the UST concentrations from 8-weekly, but not the 12-weekly dosing, could modestly discriminate clinical remission (AUC = 0.62, *p* = 0.011) (33).

In patients receiving maintenance UST every 12 weeks who experienced loss of response, 55% of cases recaptured clinical response after an escalation of the dose to every 8 weeks, suggesting a dose–response relationship (35, 36). Compared with anti-TNF-exposed patients, anti-TNF-naive patients showed higher clinical remission with UST. However, the UST concentrations in the serum were comparable in both groups (35, 36).

STARDUST, a phase 3b randomised trial, compared UST therapeutic strategies in patients with CD using early endoscopic assessment and treat-to-target *versus* standard of care, demonstrating broadly similar serum UST concentrations between groups out to week 48 (37). Of note is that the serum UST concentration was not used as a treat-to-target criterion. In the long-term extension study to week 108, the median UST concentration remained broadly similar between groups (2.28 *vs*. 2.20 μg/ml), with no significant difference in the endoscopic response (37). Stratified serum UST concentrations were not provided based on a dosing frequency of 4-weekly, 8-weekly, or 12-weekly.

Observational trials have reported associations between the serum UST concentration and a range of outcomes. A prospective Dutch registry associated higher UST trough concentrations at weeks 8 and 16 with higher odds of biochemical remission, with the greatest effect for serum UST concentration >6.3 μg/ml (38). In a Canadian cohort (*n* = 62), UST levels >4.5 μg/ml at 26 weeks were associated with significantly higher rates of endoscopic response compared with UST levels <4.5 μg/ml (39). Similarly, UST levels >4 ug/ml were associated with biochemical and endoscopic response in a cohort of 177 patients with IBD, although endoscopic response was only reported for eight participants (40). For both studies, the results were not linear across quartiles and were not replicated in the rates of clinical response/remission. Higher UST concentrations were observed in a retrospective cohort of patients with CD, reaching a composite endpoint of Simple Endoscopic Score (SES)-CD ≤5 and faecal calprotectin <150 μg/g. However, this SES-CD metric does not align with the current definitions of mucosal healing (SES-CD ≤ 2), and there were up to 13 months between the measurement of UST levels and the endoscopic evaluation for some participants (41). Whilst these observational trials suggest a signal of association between higher UST drug levels and relevant endpoints, the studies are frequently limited by small populations, incomplete data, and temporal disconnection between the measurement of UST levels and the outcome measure. Whilst the majority of these observational studies offered UST concentration thresholds to reach their clinical endpoint, the high risk of bias from the heterogeneity in the IBD phenotype, past treatment history, and variation in commercial assay use limits their translatability across IBD populations.

Dose escalation or reinduction dosing and its association with recapturing the clinical response have been investigated by several cohort studies. A systematic review of 15 cohort studies of 925 UST-treated patients with CD who underwent dose escalation reported that more than 50% of patients were able to capture response and that 40% of patients achieved corticosteroid-free clinical remission (42). In addition, 61% of patients achieved endoscopic response, and 29% achieved endoscopic remission. Dose interval shortening to every 4–6 weeks was the most frequent escalation strategy used in these studies.

In the POWER study, 215 patients with CD who had loss of response to UST were randomised to receive either a single IV reinduction or continuation of 8-weekly SC UST. The primary endpoint of clinical response [≥100-point decrease from the baseline Crohn’s Disease Activity Index (CDAI) score or CDAI <150] at week 16 was not met. However, patients who received IV reinduction demonstrated greater biochemical normalisation and higher endoscopic remission at week 16 (43). Notably, the trial included patients who were highly refractory to treatment, with a mean disease duration of 14.4 years, and with over half of patients having a history of an inadequate response or intolerance to two or more biologics prior to UST treatment. Biologic-naive patients and those with one or two prior biologics before UST and patients with a high inflammatory burden benefitted from IV reinduction, whilst improvement with IV reinduction was most modest in patients with a history of three or more prior biologics before UST (43).

The REScUE multicentre, randomized, placebo-controlled trial randomised adults with moderate–severe CD 1:1 to receiving a single IV reinduction with UST ~6 mg/kg followed by either SC UST 90 mg every 4 weeks or every 8 weeks until week 48. Among patients experiencing secondary loss of response to UST, dose intensification with a single IV administration followed by 4-weekly SC dosing of UST was not more effective than a single IV administration followed by 8-weekly SC dosing of UST (44). The median UST level at baseline was below 2 μg/ml in both groups. After IV reinduction, an increase in the UST levels of approximately 20 μg/ml was observed at week 4 in both groups. At week 8, the median serum levels of UST were 10.8 μg/ml (IQR = 8–15.9 μg/ml) and 8.2 μg/ml (IQR = 4.9–11.1 μg/ml) in the 4-weekly group and the 8-weekly group, respectively (*p* = 0.002). At week 48, although the serum levels remained significantly higher in the 4-weekly group, the median serum level of UST in the 8-weekly group remained >50% higher compared with the baseline. The UST serum levels at week 48 were 6.0 μg/ml (4.8–9.6) and 2.1 μg/ml (1.6–2.9) in the 4-weekly group and the 8-weekly group, respectively (*p* < 0.001). There was no significant correlation between the serum UST levels and steroid-free clinical remission, endoscopic remission, or clinical remission at week 48. Of interest is that a target UST trough level of 1.4 μg/ml was associated with clinical remission (33). In REScUE, both treatment groups were above >2 μg/ml through week 48. This challenges the perception that a general target trough concentration for UST exists and suggests that pharmacokinetic–pharmacodynamic modelling-based dosing may be more informative (45).

### Ustekinumab therapeutic drug monitoring in ulcerative colitis

The UNIFI trial assessed the use of UST as induction and maintenance therapy in patients with moderate to severe UC. At 8 weeks after induction, the serum UST concentrations were threefold higher in participants receiving 6 mg/kg induction compared with 130 mg, suggesting a linear relationship between dose and serum concentration (46, 47). At week 8, each quartile of serum UST concentration led to an incremental increase in the proportion of patients with clinical response, from 41% in Q1 to 74% in Q4 (47). Similar improvements were observed in the rates of clinical remission at weeks 24 and 44, and an UST concentration of ≥3.7 μg/ml was a modest discriminator of clinical remission (AUC = 0.635) and endoscopic response (AUC = 0.632) at week 44 (46).

In a real-world cohort of biologic-experienced UC patients, the week 16 median serum UST trough levels were significantly higher in those with week 16 endoscopic response (5.0 *vs*. 2.4 μg/ml) and endoscopic remission (6.0 *vs*. 2.1 μg/ml) (48).

An exposure–response relationship may be present between the steady-state serum UST trough concentrations and the outcome measures in IBD. However, given the wide variation in the suggested therapeutic trough concentrations, coupled with growing evidence of the lack of benefit from dose escalation in UST, regular monitoring of the serum UST concentration is not currently recommended in IBD.

### Relationship between ustekinumab concentration and patient safety in IBD

Evidence does not suggest an association between the concentration of UST and the rates of infection or other adverse safety events (33, 46). Biosimilar UST therapies are likely bioequivalent to reference UST (49, 50). UST concentrations of 3–7 µg/ml at week 8 and 1–3 µg/ml during maintenance have been associated with improved clinical outcomes. Dose optimisation may benefit those with a partial response or loss of response to standard dosing. Based on the results from recent large randomised controlled studies (POWER and REScUE), it may seem prudent to switch to another advanced treatment if there is no documented clinical effect within 24 weeks of IV reinduction with UST. Whether serial IV reinduction may also be an option with repeated loss of response requires further study.

### Ustekinumab anti-drug antibodies

UST anti-drug antibodies are detectable. However, in IM-UNITI, incidence was low (2.3%) across the 44-week study period, often transient, and did not strongly correlate with the serum UST concentration (33). Interestingly, antibodies were more likely to be detectable in induction responders who entered IM-UNITI in the placebo arm (5.3% *vs*. 2.9%) (33). There are no available published data on the incidence of anti-drug antibodies in non-responders of the UNITI induction trials.

### Proactive therapeutic drug monitoring with ustekinumab

There are currently no prospective randomised controlled trials (RCTs) examining proactive TDM in IBD. A retrospective single-centre cohort study attempted to stratify patients with IBD into proactive or reactive TDM groups and described an association of proactive TDM with drug persistence and avoidance of IBD-related hospitalisation (51). Well-designed prospective RCTs are required to understand the role of proactive TDM across advanced therapies in IBD.

### IL-23 p19 monoclonal antibodies

#### Risankizumab therapeutic drug monitoring

Risankizumab (RZB) is a monoclonal antibody that specifically targets the IL-23 p19 subunit. It demonstrated efficacy in the induction and maintenance of remission in moderate to severe CD in the ADVANCE/MOTIVATE trial (induction) (52) and FORTIFY trial (maintenance) (53). Its efficacy in moderate to severe UC was demonstrated in the INSPIRE and COMMAND trials (54). RZB achieves steady-state concentrations 16 weeks after the standard induction regimens. Its half-life is between 21 and 28 days in both CD and UC (55).

The clearance of RZB in CD is affected by female sex, high baseline body weight, CRP level, albumin, and prior advanced therapy exposure, whilst that in UC by high baseline body weight and low albumin, which increase drug clearance. However, the impact is small and not clinically relevant to RZB dosing (55, 56). In CD and UC trials, the serum RZB concentrations were broadly dose proportional, and antibody formation was low (2%–4%). In CD, the RZB trough concentrations were impacted by body weight and the serum albumin level, without impact on the clinical outcomes, and there was little pharmacokinetic benefit from increasing the daily dose to 1,200 mg (55). In CD, higher quartiles of serum RZB levels were not associated with incremental improvements in the clinical or endoscopic response at week 52, nor incremental adverse events (55). In UC, daily dosing of 1,200 mg had the best exposure–response relationship, with little added benefit from higher dosing (56).

Published data exploring serum drug levels and clinical, biochemical, and endoscopic outcomes are limited. A small prospective study of 28 patients with CD found that the mean maintenance trough concentrations of RZB were significantly higher at 18 months in patients in clinical and biochemical remission compared with those not in remission (21.6 ± 13.3 *vs*. 7.4 ± 6.4 µg/ml, *p* = 0.001). A dose–response effect was observed, with increasing RZB trough concentrations being associated with higher rates of remission. Receiver operating characteristic (ROC) curve analysis (AUC = 0.93, *p* < 0.001) identified a maintenance trough level above 11.5 µg/ml as significantly associated with clinical and biochemical remission (sensitivity = 81.8%, specificity = 80.3%) (57).

In a subgroup analysis of the FORTIFY (phase 3, double-blind, re-randomised responder withdrawal) maintenance study, patients with an initial response to the standard 12-week IV induction were re-randomised to RZB 180 mg SC, RZB 360 mg SC, or placebo every 8 weeks for 52 weeks. From week 16, patients with inadequate or loss of response were eligible for rescue therapy (single dose of RZB 1,200 mg IV, followed by RZB 360 mg SC every 8 weeks). Administration of this ‘rescue’ treatment recaptured clinical remission and/or endoscopic response in 20%–36% of patients who had inadequate response to RZB (58).

Observational RZB dose escalation studies are beginning to emerge. A retrospective study of 20 patients with IBD with an inadequate response to RZB maintenance therapy demonstrated that dose escalation to 360 mg SC every 4, 6, or 7 weeks resulted in improvement in the clinical symptoms in 70% of patients, with associated improvement in the laboratory parameters (haemoglobin and albumin) and body weight (59, 60). In another cohort of 17 CD patients with inadequate or loss of response to RZB, amongst those who underwent dose intensification to 360 mg SC every 4 or 6 weeks, 71% showed clinical response, 53% achieved steroid-free clinical remission, and 67% demonstrated endoscopic improvement (60).

Finally, in a retrospective study of 12 patients with clinically or endoscopically active CD, dose escalation of RZB achieved symptomatic improvement in 50% (six patients) (61).

Taken together, early evidence suggests a potential role for TDM and dose optimisation with RZB. Larger prospective, well-designed dose optimisation trials with TDM are now needed.

#### Mirikizumab therapeutic drug monitoring

Mirikizumab (MIRI) is a humanised IgG4 monoclonal antibody targeted against the p19 subunit of IL-23. LUCENT-1 and LUCENT-2 demonstrated its efficacy in moderate to severe UC (62), whilst VIVID-1 showed its efficacy in CD (63). As with other monoclonal antibodies, the pharmacokinetics of MIRI are impacted by body weight and albumin concentration, below the threshold required for dose adjustment (64). It has a half-life of 9.5 days (64, 65). In the LUCENT trials, 23% of MIRI-treated patients developed ADAs, but only 2.6% of these patients exhibited reduced drug levels, with negligible clinical effects (62).

In CD, the average MIRI concentration was no different between endoscopic responders and non-responders at week 12 (66). There was a non-significant trend towards slightly higher adverse events with increased drug exposure, but with the adverse events not directly correlated with the serum MIRI concentration (66). LUCENT participants with loss of response during maintenance were reinduced with IV MIRI; however, the number of this subpopulation was too low (*n* = 10) to determine the impact of MIRI concentration on response (67). There are currently insufficient data to recommend a role for TDM with MIRI.

#### Guselkumab therapeutic drug monitoring

Guselkumab (GUS) is a fully human IgG1λ monoclonal antibody against IL-23 p19 that has been recently approved for IBD (68, 69). The pharmacokinetics of GUS are impacted by body weight, serum albumin, CRP, age, sex, and prior biologic failure status, which are statistically significant factors in drug clearance, but with no clinically meaningful effects and therefore not needing dose adjustment (70). It has a half-life of approximately 16.5 days. In the pivotal trials, the IV induction dose of 200 mg at weeks 0, 4, and 8 demonstrated near maximal efficacy with no additional benefit from larger doses (71, 72). In CD, induction dosing of 400 mg SC every 4 weeks for three doses significantly improved the clinical and endoscopic outcomes at week 12 *versus* placebo (68). Similar evidence for SC induction dosing of 400 mg every 4 weeks for three doses was demonstrated with improved clinical outcomes (73).

In the GALAXI-2 and GALAXI-3 trials for CD, antibodies to GUS developed in 5% of patients in both the 200- and 100-mg maintenance groups through week 48 (74). Although neutralising antibodies were present in 13% of the antibody-positive patients in the 100-mg group and none in the 200-mg group, the efficacy outcomes were similar (74). In the QUASAR studies for UC, 1.4% of all GUS-treated patients were noted to have neutralising antibodies, with no impact on the serum concentration, efficacy, or safety (69). TDM data for GUS in IBD are not available, with the current dosing regimens achieving maximal efficacy. There is currently no role for TDM with GUS.

### JAK inhibitors

JAK inhibitors are a family of small molecules that block one or more of the intracellular tyrosine kinases, including JAK-1, JAK-2, JAK-3, and TYK-2 (75). Compared with biological drugs, JAK inhibitors can be administered orally, have a short half-life and rapid onset of action, and no immunogenicity. Tofacitinib (TOFA) is an oral small molecule that inhibits all four of the JAK family of tyrosine kinases, with a preference for JAK1 and JAK3 inhibition, leading to the modulation of multiple cytokine pathways (75). It has a half-life of approximately 3 h. The efficacy of TOFA for the induction and maintenance of remission in moderate to severe UC was demonstrated in the OCTAVE studies (76). TOFA is rapidly absorbed, with peak concentrations achieved within 1 h of administration, and the plasma concentration is unaffected by disease severity (76). There were no differences in the plasma TOFA concentrations when stratified by remission status at week 8 or week 52 (76). The TOFA levels remained stable throughout induction and maintenance (77).

Filgotinib (FILGO) is an oral selective JAK1 inhibitor. It has a half-life of approximately 5 h and is rapidly absorbed, achieving plasma concentrations within 1–5 h. FILGO is metabolised to form an active JAK1-selective major metabolite; as such, there is no pharmacodynamic gain from increasing the dose beyond 200 mg daily (78). FILGO can be used for induction and maintenance of remission in moderate to severe UC, as demonstrated by the SELECTION trial (79). In this trial, the FILGO concentrations were similar across responders and non-responders in both the induction and maintenance studies, and safety events were not associated with higher FILGO concentrations (79).

Upadacitinib (UPA) is a selective JAK1 inhibitor. U-ACHIEVE and U-ACCOMPLISH demonstrated the efficacy of UPA in the induction and maintenance of remission in moderate to severe UC (80), with similar outcomes from U-EXCEL, U-EXCEED, and U-ENDURE in moderate to severe CD (81). UPA achieves peak plasma concentrations within 1–2 h and has a half-life of approximately 8–14 h (82).

There is no clear role for TDM with JAK inhibitors in clinical practice. With UPA, maximal clinical and endoscopic benefits were achieved at induction dosing of 45 mg daily, followed by maintenance dosing at 30 mg daily, with data suggesting that extended induction to 16 weeks may improve the clinical response in those who did not achieve an adequate response with 8 weeks of induction (83). Similarly, for FILGO, the phosphorylation of JAK-related IL-6-induced STAT-1 plateaued at licensed maintenance doses of 200 mg daily (84).

### Sphingosine-1-phosphate receptor modulators

Sphingosine-1-phosphate (S1P) receptor modulators are a newer class of small-molecule medications for UC (85). Ozanimod is an oral S1P receptor modulator that primarily binds to the S1P1R and S1P5R subtypes and is used for the induction and maintenance of remission in moderate to severe UC, supported by data from the TRUE NORTH trial (86). There are no data for ozanimod concentration related to clinical response or toxicity. Like FILGO, ozanimod is metabolised to several major active metabolites including CC112273 (87). The pharmacokinetics of ozanimod are similar in the licensed indications of UC and multiple sclerosis (88). Whilst the pharmacokinetics of ozanimod can be influenced by body weight and smoking status, this is not considered sufficiently significant to the dose by weight (88), and ozanimod exposure is not affected by high-fat diet (89). Its half-life is 21 days; however, with active metabolites CC112273 and CC1084037, the half-life may be longer at between 84 and 117 h (90). There are no data for TDM with ozanimod.

Etrasimod is a once-daily oral synthetic S1PR1, S1PR4, and S1PR5 modulator, with higher selectivity for S1PR1 than for S1PR4 and S1PR5 (85). It demonstrated efficacy in the induction and maintenance of remission in moderate to severe UC, as per the ELEVATE trial (91). The pharmacokinetics are approximately linear, with steady state reached after 7 days of regular dosing, and dose-dependent relative lymphopenia was observed up to tested doses of 3 mg (92). Its half-life is approximately 30 h. There is currently no role for etrasimod TDM. The currently proposed therapeutic drug concentration thresholds and associated clinical outcomes across non-anti-TNF advanced therapies are summarized in **Table 2**.

**Table 2:** Summary of the trough concentrations and targets associated with outcomes.

Drug	Timing (induction/maintenance)	Trough concentration	Outcome	TDM guidance
Vedolizumab	Week 6; induction Week 14; maintenance	Week 6: 33–37 µg/ml Week 14: 15–20 µg/ml Maintenance: 10–15 µg/ml	Week 6 (>37.1 µg/ml): associated with higher rates of clinical remission (*post-hoc* GEMINI; Rosario) Week 6 (>30 µg/ml): associated with higher clinical remission at weeks 14 and 52 (ERELATE) Week 14 (~18.4 µg/ml): proposed target concentration associated with improved outcomes (*post-hoc* GEMINI; Rosario) Maintenance (~12.7 µg/ml): proposed target concentration associated with improved outcomes (*post-hoc* GEMINI; Rosario) Dose optimisation at week 6 compared with standard dosing did not improve clinical or endoscopic response at week 30 (ENTERPRET)	Routine TDM is not recommended
Ustekinumab	Week 8; induction Weeks 16–26; early maintenance Week 44; maintenance	Week 8: 3–7 µg/ml (synthesised) Week 16: 5–6 µg/ml (observational) Week 26: >4.5 µg/ml Week 44: ≥3.7 µg/ml Maintenance: 1–3 µg/ml (synthesised)	Higher clinical remission observed with 8-weekly compared to 12-weekly dosing in UNITI-2 Week 8: Increasing concentrations associated with increased rates of clinical response (quartile-based analysis, UNIFI) Higher ustekinumab concentrations associated with increased clinical remission at week 24, particularly with 8-weekly dosing (IM-UNITI) Week 16: (5–6 µg/ml): higher levels observed in patients with endoscopic response/remission (observational) Week 26: (>4.5 µg/ml): associated with higher endoscopic response (observational) Week 44 (≥3.7 µg/ml): modest discrimination of clinical remission and endoscopic response (UNIFI; Adedokun et al.) Maintenance (1–3 µg/ml): associated with improved clinical outcomes (synthesised) No significant correlation between serum ustekinumab levels and clinical remission, steroid-free clinical remission, or endoscopic remission at week 48 (REScUE) Dose escalation or reinduction may recapture clinical response in >50% of patients, with reported improvements in endoscopic response and remission.	Routine TDM is not recommended
Risankizumab	Induction (to steady state) Maintenance (steady state ~16 weeks post-induction)	Maintenance >11.5 µg/ml (small prospective study)	No association between higher risankizumab concentrations and clinical or endoscopic response across quartiles in Crohn’s disease phase 3 trials In UC, exposure–response analysis suggests minimal additional benefit with increased dosing Higher trough concentrations observed in patients achieving clinical and biochemical remission in a small prospective study (21.6 *vs*. 7.4 µg/ml), with a proposed threshold >11.5 µg/ml Dose escalation may improve clinical outcomes in cohort studies, although no corresponding serum concentration data available Overall, limited evidence for exposure–response relationship and limited data to support routine TDM at present	Routine TDM is not recommended
Mirikizumab	Week 12; induction Maintenance	–	No significant difference in serum mirikizumab concentrations between endoscopic responders and non-responders at week 12 in Crohn’s disease No significant correlation between serum mirikizumab concentration and adverse events Insufficient data to determine the impact of drug levels on response following reinduction Overall, insufficient evidence to support a role for TDM	Routine TDM is not recommended
Guselkumab	Induction Maintenance	−	No data available to evaluate the relationship between serum guselkumab concentration and clinical/endoscopic outcomes No additional efficacy observed with higher dosing in clinical trials Anti-drug antibodies occur at low frequency and do not impact the serum concentration, efficacy, or safety. Overall, no evidence to support a role for TDM	Routine TDM is not recommended
Tofacitinib	Week 8; induction Week 52; maintenance	−	No difference in the plasma tofacitinib concentrations between patients achieving remission and those not in remission at week 8 or week 52 Tofacitinib concentrations remain stable across induction and maintenance No immunogenicity associated with tofacitinib and no role for TDM	Routine TDM is not recommended
Filgotinib	Induction Maintenance	−	Filgotinib concentrations were similar between responders and non-responders in both induction and maintenance studies (SELECTION) Overall, no evidence to support a role for TDM	Routine TDM is not recommended
Upadacitinib	Induction Maintenance	−	No evidence evaluating the relationship between upadacitinib concentrations and clinical or endoscopic outcomes Overall, no clear role for TDM in clinical practice	Routine TDM is not recommended
Ozanimod	Induction Maintenance	−	No data evaluating the relationship between ozanimod concentrations and clinical or endoscopic outcomes No data to support a role for TDM	Routine TDM is not recommended
Etrasimod	Induction Maintenance	−	No data evaluating the relationship between ozanimod concentrations and clinical or endoscopic outcomes No data to support a role for TDM	Routine TDM is not recommended

TDM, therapeutic drug monitoring; UC, ulcerative colitis.

## Conclusion

The exciting advent of novel therapeutics in IBD raises the interesting question of how to maximise their effectiveness for patients. TDM has shown its utility in IBD, with monitoring of thiopurines and with anti-TNF biologics. As discussed in this review, the data for TDM in non-anti-TNF advanced therapies for IBD are currently scarce, and universally accepted therapeutic trough concentration targets are yet to be established for non-anti-TNF advanced therapies. TDM for non-anti-TNF advanced therapies is not currently recommended. The relatively stable, linear pharmacokinetics of small-molecule therapies such as JAK inhibitors and S1P receptor modulators mean that TDM is unlikely to have a role. However, for the immunoglobulin-based monoclonal antibodies, particularly VDZ where the evidence for an exposure–response relationship is most consistent, it is biologically plausible that TDM could help clinicians optimise drug concentrations, in addition to understanding whether non-response is due to a low drug concentration, an antibody formation, or a class failure. However, the current burden of evidence only associates the drug concentration with a range of clinical outcomes. This is not causation, and the incorporation of TDM on this basis risks overinterpreting its usefulness. To realise the potential of TDM and perhaps realise more personalised medicine, well-designed randomised trials are required. This is particularly relevant given that people with more active IBD-related inflammation may sequester more of the active drug, leading to faster drug clearance and lower measurable serum concentrations. Therefore, IBD activity is a key confounder that leaves observational studies open to overreporting the inverse relationship between drug levels and clinical activity.

There are practical questions to answer for future research, including the optimal timing for drug concentration monitoring and standardisation of the drug concentration reporting between commercially available assays. Retrospective trials are often unable to capture the lag between TDM request and result, which can result from delays along the pathway from patient attendance through laboratory processing to clinician decision making. TDM pathways themselves may impact the effectiveness of TDM by affecting timely dose adjustment and therapeutic decisiveness, particularly for a proactive TDM approach (93). The implementation of TDM using a proactive or reactive approach requires further study.

Further work focused on large, well-designed randomised trials is required to determine whether TDM has a role in daily clinical practice for patients on non-anti-TNF advanced therapies.
